# Strategies and Recommendations to Improve Accessibility of Essential Surgery in Rural Settings in OECD Countries: A Scoping Review

**DOI:** 10.1002/wjs.12631

**Published:** 2025-05-31

**Authors:** G. Osman, Y. Kamel, I. Konate, M. Diedhiou, S. Basnet, R. Shrestha, S. K. Shrestha, V. Krylyuk, J. Grushka, E. Wong, T. Farhat, K. Khwaja, D. Deckelbaum

**Affiliations:** ^1^ Centre for Global Surgery McGill University Montreal Canada; ^2^ Faculty of Medicine University of Montreal Montreal Canada; ^3^ Faculty of Health Sciences Université Gaston Berger Saint‐Louis Sénégal; ^4^ Dhulikhel Hospital Kathmandu University Dhulikhel Nepal; ^5^ Kyiv Hospital for Emergency Medicine (KHEM) Kiev Ukraine; ^6^ Faculty of Medicine Division of General Surgery McGill University Montreal Canada

**Keywords:** essential surgery, global surgery, remote, rural

## Abstract

**Background:**

The provision of essential and emergency surgical services presents complex challenges in remote areas. Equitable access has gained attention thanks to the significant work done by the Lancet Commission on Global Surgery (LCoGS). Although the focus was on low‐ and middle‐income countries, developed countries also face challenges in providing equitable surgical care and, in fact, do not always meet the benchmarks set by the LCoGS yet still have acceptable outcomes. We sought to explore the current strategies aimed at improving and maintaining access to essential surgical care in rural and remote areas of OECD countries (Organization for Economic Co‐operation and Development).

**Methods:**

We conducted a scoping review using MeSH terms. The search was performed on MEDLINE and EMBASE databases and was limited to English sources published between 1946 and January 10, 2025.

**Eligibility criteria:**

Any strategy or intervention aimed at improving and maintaining timely access to essential surgeries in rural and remote areas of OECD countries.

**Results:**

Six main categories of strategies were found: (1) resource distribution; (2) task sharing; (3) telemedicine; (4) surgical workforce; (5) training and education; and (6) prehospital system.

**Conclusion:**

Recognizing that developed countries, in fact, do not always meet the benchmarks set by the LCoGS yet still have acceptable outcomes highlights that specific strategies are important contributors to the reduction of disparities between rural and urban outcomes. These strategies may be used in the study of surgical services in low‐ and middle‐income countries.

## Graphical abstract

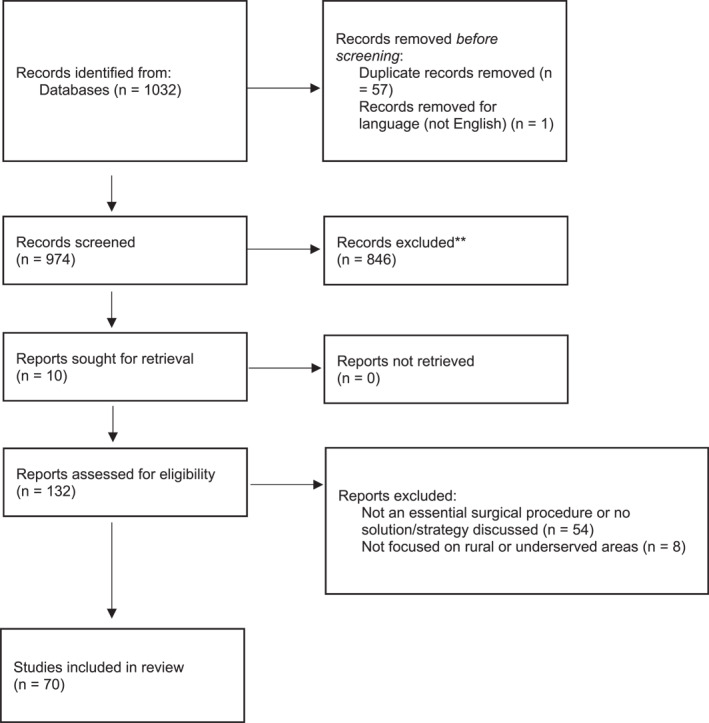

## 1 Introduction

The Lancet Commission on Global Surgery (LCoGS) is approaching its 10th anniversary because it was first published in 2015 [1]. With the 68th World Health Assembly adopting the resolution that access to essential surgery and anesthesia are integral to universal health coverage, global surgery has become an increasingly growing field to support surgical systems in low‐ and middle‐income countries (LMICs) [2]. The LCoGS has identified six indicators to improve surgical care: access to timely essential surgery, specialist surgical workforce density, surgical volume, perioperative mortality, protection against impoverishing expenditure, and protection against catastrophic expenditure. Considering that LMICs are more affected by gaps in surgical care, their provision of surgical care was the main focus of the Lancet Commission [1]. However, it is well documented that high‐income countries face similar challenges in providing surgical care to the entirety of their populations. For years, a shortage of general surgeons in high‐income countries has been forecast, mostly affecting rural and underserved areas [3, 4]. In addition to the predictable problem of increased distance to a hospital, rural and remote areas of high‐income countries (HICs) face numerous challenges in access to surgical care. Notably, well‐documented issues such as financial constraints and limited resources remain a challenge, which often may lead to rural hospital closures [5, 6, 7]. Case volume also presents a challenge in rural hospitals with the relationship between case volume and maintenance of surgical competency [8, 9, 10, 11]. Despite these issues, HICs and Organization for Economic Co‐operation and Development (OECD) countries such as Canada have more acceptable outcomes than their lower/middle‐income counterparts [12, 13, 14]. This is the result of several strategies such as regionalization of care, hospital designation, and development of mature transfer mechanisms including ground and air ambulance programs, task shifting, and education collaboratives to enhance knowledge of resuscitation strategies in preparation for safe transfer [15, 16, 17, 18, 19].

The objective of this scoping review is to explore the current strategies aimed at improving and maintaining access to essential surgical care in rural and remote areas of HICs while drawing connections with LMICs. OECD countries were used as a surrogate for HICs. The rationale behind focusing our study on OECD members is that the majority of these countries have a very high Human Development Index (HDI) [20, 21]. The HDI is a measure of a country's health, education, and income [20]. Hence, to evaluate the delivery of essential surgeries in developed health care systems, we decided to use OECD countries as the main study population. We hypothesize that many of these countries, which struggle with remote populations and access to care, rely on strategies such as augmenting the prehospital system, rapid identification of need for transfer, resuscitation sciences training, and effective transfer as effective measures to maintain appropriate outcomes. These measures are prioritized over the onsite delivery of definitive care to provide timely service to their remote populations. Therefore, a look at tools and strategies used in OECD countries to improve and maintain surgical delivery can be helpful in examining the disparities between HICs and LMICs in providing adequate surgical care to their populations.

## 2 Methods

We conducted a scoping review using PRISMA‐ScR to identify strategies used by OECD countries to provide surgical care to their rural populations. The search was performed on MEDLINE and EMBASE databases and was limited to English sources published between 1946 and January 10, 2025. Search results were screened by two independent reviewers, and discrepancies were discussed until consensus was reached. When necessary, a decision was made with a third reviewer.

### 2.1 Inclusion Criteria

The inclusion criteria of this study are any strategy or intervention aimed at improving and maintaining timely access to essential surgeries in rural and remote areas of OECD countries. OECD countries were used as a surrogate for HICs for the purpose of this paper (although few OECD countries are middle‐income, none of those appeared after entering our search criteria). Essential surgeries include any Bellwether procedure, defined by the LCoGS as emergency caesarean, emergency laparotomy, and operative management of open long‐bone fracture [1]. To have broader results and to be more inclusive of other essential surgeries, we opted to also include acute surgical needs, for example, any trauma requiring emergency surgery. Sources of evidence were open to all and include gray literature, peer‐reviewed articles, commentary, letter, web‐based articles (e.g., WHO documents, World Bank), or government/official documents.

Inclusion criteria are as follows:

- Discusses strategies or solutions to improve or maintain essential surgical access in rural and underserved areas.
- Essential surgeries include Bellwether procedures (i.e., emergency caesarean, emergency laparotomy, and operative management of open long‐bone fracture) and acute surgical needs (i.e., trauma, emergency surgeries).
- OECD countries
- English sources published between 1946 and January 10, 2025.

After full‐text screening, recurrent and similar strategies were grouped under categories and descriptors. Repeated keywords and the focus of papers were used to categorize the studies under the corresponding descriptor. For example, when a strategy involved the improvement or the implementation of a rural component in the surgical residency, this was grouped under “training and education.” Examples of keywords for this group include “surgical training,” “residency,” “rural training,” etc. These categories are elaborated in greater detail in the results section. The search was limited to OECD countries. Based on the results, studies that fulfilled the inclusion criteria were retained. As a secondary outcome, surgical outcomes of different surgical systems were extracted based on relevance to the type of paper and information availability. These included time to definitive care, distance traveled to care, complication rates, improved surgeon retention, mortality and morbidity rates, and length of hospital stay.

## 3 Results

### 3.1 Data Extraction and Categorization

**FIGURE 1:**
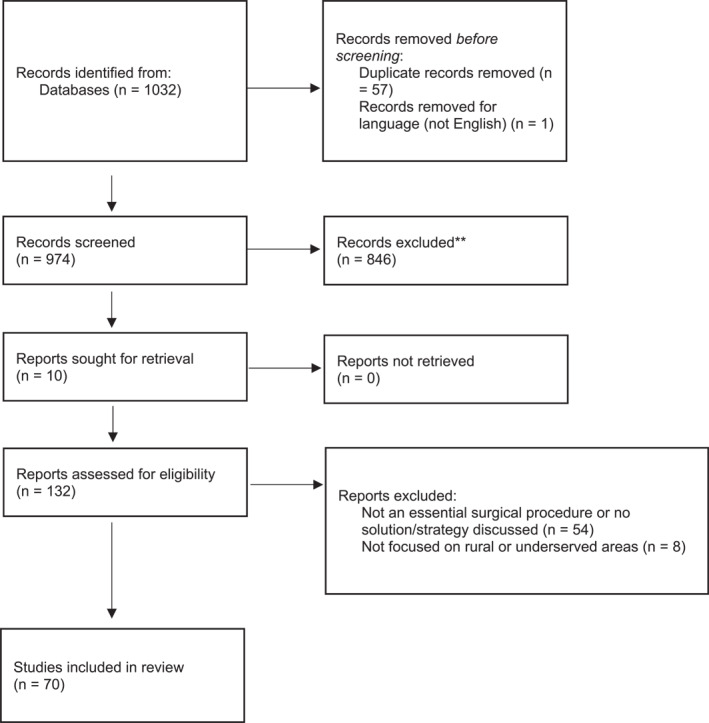
Literature search flowchart.

**FIGURE 2:**
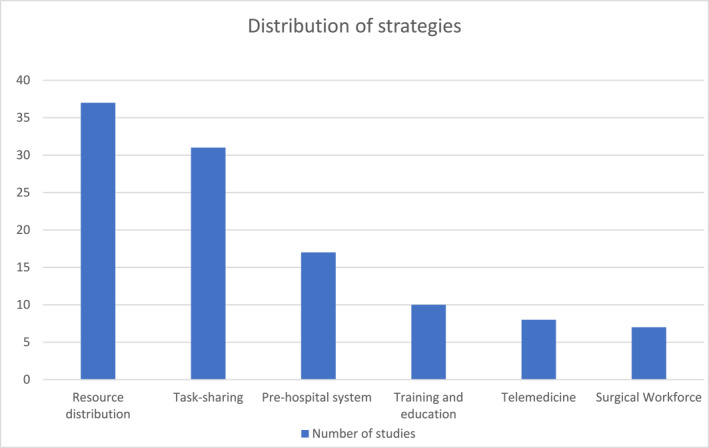
Distribution of strategies.

**FIGURE 3:**
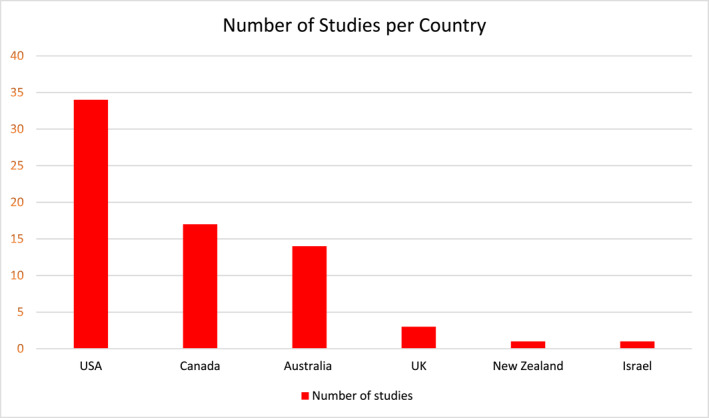
Studies by country.

After the first screening of titles and abstracts, 132 of 974 studies were retained. Seventy studies were included after full‐text screening. Of the 70, eight were between 1988 and 2000, and were included in the spirit of a comprehensive study. Figure 1 depicts the literature search flowchart. Six main categories were created to group the different focuses of strategies used by OECD countries, which are as follows: (1) resource distribution; (2) task sharing; (3) telemedicine; (4) surgical workforce; (5) training and education; and (6) prehospital system. Table 1 summarizes these strategies. These categories were chosen to regroup different strategies into one subgroup. The resource distribution subgroup included strategies that focused on improving hospital designation and resource availability by region. Task sharing included any procedure that was performed by either a nonspecialist or a nonsurgical clinician. Telemedicine regrouped strategies that used technological means (e.g., videoconference tools, telephone, teleimagery, etc.) to provide care to remote areas or improve communication between specialists and clinicians in rural and remote areas. Surgical workforce encompasses strategies that focus on the physician density in rural areas. The training and education subgroup includes strategies aimed at improving surgical trainees' abilities in providing rural care as well as potentially influencing their decisions on rural practice. This also grouped training and education of general practitioners and other nonphysician clinicians such as nurses and hospital staff. Finally, the prehospital system subgroup encompasses strategies aimed at improving the prehospital care such as transportation from injury sites or during interhospital transfers (e.g., air transport, ambulance, emergency response). It is important to note that these strategies are not mutually exclusive, as one strategy can touch on two or more categories. For example, an emergency response to a trauma case that requires a transfer to a central hospital would touch both the prehospital system and resource distribution. Therefore, a paper may cover more than one strategy. Figure 2 shows the distribution of strategies used by different countries, and Figure 3 depicts studies by country.

**TABLE 1:** Strategies used to improve and maintain surgical care in remote and low‐resource settings.

Strategy	Description	Evidence‐based recommendations
Resource distribution	Focus on improving the hospital location and resource availability by region, based on the type of services provided.	Study optimal resource distribution for local health care delivery. Based on resources, evaluate the cost‐effectiveness and efficiency for regionalized versus decentralized care provision in a specific context. Regionalized trauma care and regionalized ACS may improve patient outcomes [15, 19, 22, 23, 24, 25, 26, 27, 28].
Prehospital system	Strategies that improve prehospital care such as transportation from the injury site in trauma cases or during interhospital transfers.	Optimize hospital designation and transfer of acutely ill or injured patients. Improve transportation means and implement a structured plan for rapid on‐site response and transfer [19, 24, 29, 30, 31, 32, 33].
Task sharing	Provision of a service that was performed by either a nonspecialist or a nonsurgical clinician	Determine appropriateness and feasibility in a specific setting of developing task sharing for certain procedures that would increase their availability and timely delivery [34, 35, 36, 37].
Training and education	Strategies aimed at improving surgical trainees' abilities in providing rural care and to potentially influence their decisions on rural practice. Training and education of general practitioners and other nonphysician clinicians, such as nurses and hospital staff, in providing optimal rural surgical care.	Implementing rural surgical care fellowships and residency programs may improve the rural surgical workforce and better prepare surgeons for rural care [38, 39, 40]. Training local health care staff in providing rural surgical care may improve service delivery [40]. Training local health care staff in resuscitation sciences and early recognition of the need for transfer improves timely care provision [19].
Surgical workforce	Increase the overall surgical workforce in rural and low‐resource settings.	Strategies may include incentivization, work/life attractiveness in rural practice, and providing opportunities to attract trainees to rural practice. Early exposure to rural surgical practice and educational opportunities in medical schools and residencies may increase interest in rural practice [3, 38, 41].
Telemedicine	Strategies that make use of technological means (e.g., videoconference tools, telephone, teleimagery, etc.) to provide care to remote areas or improve communication between specialists and clinicians (generalists or nonphysician clinicians) in rural and remote areas.	Optimize local and interhospital communication between health care professionals. Evaluate the possibility and feasibility in context‐specific settings of implementing telemedicine tools (e.g., videoconferencing equipment, teleimaging equipment) [27, 32, 42].

The most prevalent focus of strategy was resource distribution, with 37 papers showing evidence of this strategy. Task sharing was the second most prevalent strategy, followed by the prehospital system, training and education, telemedicine, and surgical workforce. Studies from the United States were the most prevalent, with the country having a large number of rural areas. Canada and Australia also have a large portion of rural populations and had a similar number of studies—17 and 14, respectively. Finally, the remaining countries that had studies that met inclusion criteria were the United Kingdom, New Zealand, and Israel.

## 4 Discussion

Providing essential and emergency surgical services in remote areas is challenging. Although the LCoGS indicators highlight equitable access, interventions to improve timely, quality surgical care in LMICs remain limited [43]. More research is needed on financing, economics, and trans‐sectoral approaches in LMICs [44]. Although LCoGS identified limitations in LMICs, HICs also face challenges delivering surgical care to rural and remote areas, which are traditionally less resourced than urban centers. However, HICs generally achieve better outcomes compared to LMICs in the care of rural populations [12, 13, 14]. This review examines OECD strategies to ensure accessibility and outcomes for rural surgical patients, with resource distribution, task sharing, and prehospital interventions emerging as key focuses.

### 4.1 Current Issues in OECD and Outcome Disparity

To evaluate the context of the abovementioned strategies, it is useful to examine the current issues of OECD countries in providing equitable essential surgical care. The LCoGS recommends increasing the surgical workforce to “at least 20 surgical, anesthetic, and obstetric physicians per 100,000 population by 2030” [1]. However, this goal is challenging, even in developed countries with rural areas facing shortages, such as the United States and Canada [3, 4, 39, 45]. Notably, the general surgeon density in the remote territory of Nunavut, Canada, is 3/100,000 people [46]. In the Northwest Territories, the surgical density is 15/100,000 people [46]. These statistics are strikingly close to the estimated surgical, anesthesia, and obstetric (SAO) workforce of 1/100,000 in low‐income countries and 8/100,000 in lower‐middle‐income countries [47]. Although the target numbers of the Lancet Commission are a sum of the SAO workforce, the number of general surgeons is problematic in itself in the United States, especially in rural territories, as the density per 100,000 continues to decline since the 1980s [48]. The LCoGS indicator of timely access to essential surgeries, whose target is that 80% of the population can access a facility providing Bellwether procedures within 2 h, is also problematic in HICs. For example, a Canadian report showed that, within provinces, between 2% and 30% of rural pregnant women must travel over 2 hours for delivery. The situation is markedly worse in the Yukon, Nunavut, and Northwest Territories, where 99.5%, 84.1%, and 61.9% of women, respectively, must travel over 2 hours for hospital delivery [49]. Although outcomes for women in rural areas are slightly worse than for women in urban areas, maternal mortality remains relatively rare for both groups [49]. Additionally, a survey in Quebec showed that Bellwether procedure facilities are concentrated in the southern third of the province, leaving most of the remaining two‐thirds without surgical care within 2 hours [50].

Despite these challenges, surgical outcomes in HICs remain acceptable compared to rural populations in LMICs where outcomes are strikingly worse [12, 13, 14, 51]. Although comparing outcomes directly is complex, exploring the etiology of these disparities remains pertinent. HICs have implemented strategies described below to mitigate the impact of distance, workforce limitations, and other challenges.

### 4.2 Strategies to Provide Surgical Care to Rural Populations

#### 4.2.1 Resource Distribution

Resource distribution was an important strategy to improve surgical access to remote populations. This strategy subgroup differed depending on location. Notably, in the United States, critical access hospitals (CAHs) play an integral role in providing emergency care to rural areas [52]. Regionalization was more prevalent in the context of trauma care and emergency surgery. Although a decentralized system was more commonly seen in cases of obstetric care [53], regionalization has also shown potential improvement in perinatal care [54]. Both decentralization and centralization have shown benefits in different settings [55]. However, the effects of regionalization on emergency health services have completely changed health care delivery in high‐income countries such as Canada. In Quebec, the implementation of a trauma system, which included regionalization and improving the prehospital system, has decreased trauma‐related mortality from 51.8% to 8.6% over a period of 10 years, from 1992 to 2002 [15]. Significant positive effects of the regionalization of trauma care were also seen in other OECD countries such as the United States, the United Kingdom, Australia, and the Netherlands [16, 23]. Implementing a trauma system and regionalization are important components in trauma care and improve trauma‐related mortality rates [15, 22, 23]. Regionalization is not limited to trauma surgeries and is also used in the management of acute general surgery in certain countries such as the United States [28].

Acute care surgery models also support the importance of optimizing resource distribution [56]. These models have the potential to decrease mortality [57, 58, 59], decrease morbidity [59, 60, 61], decrease length of stay [57, 62, 63], and lower costs of care [57, 60, 62]. Efficient rural outreach and essential service provision are vital. Without it, regional tertiary centers may face improper triaging and referrals, undermining system efficiency [64]. Thus, effective identification and transfer of patients that supersede local capacity are crucial for regionalized systems to function effectively. These strategies have also been shown to be effective in LMICs [65].

#### 4.2.2 Task Sharing

Task sharing involves nonspecialists, general practitioners, or non‐physician clinicians assuming surgical or anesthetic tasks [1, 34, 66]. Hernia repairs [67], appendectomies by GP‐surgeons [35], GP‐provided anesthesia, and GP‐provided operative delivery [34, 36] are commonly used examples of task sharing. In the United States, certified nurse anesthetists played an important role in contexts of obstetric care and emergency trauma cases [66, 68]. In emergency obstetrics in the United States and Canada, this strategy was commonly used and found to be a safe and effective alternative to specialist‐provided services [69].

Although task sharing may surely increase access to certain surgical services for rural populations, it remains a limited strategy considering the scope of practice and represents only a small fraction of surgical systems. However, it may represent a strategy to improve early recognition of the need to transfer through improved knowledge acquisition using existing transfer corridors. In the context of LMICs, this strategy is not considered sufficient to meet the needs in essential surgery and anesthesia [1].

#### 4.2.3 Prehospital System

The prehospital system is a crucial component to provide effective surgical care. It is estimated that 54% of deaths in LMICs may be addressable by prehospital and emergency care, and an estimated 24% of these deaths are surgically treatable [70]. The rapid identification, alert, and transport of a patient to the nearest hospital are clearly crucial for their outcome. Although many HICs rely on robust prehospital systems, including air transfer and sometimes mobile teams, these may not be feasible in the near term in LMICs [71, 72]. Important strategies such as education and training to support prehospital personnel in both basic and advanced resuscitative measures have been demonstrated to have a positive impact in both HICs and LMICs [1, 18]. In fact, significant improvements in mortality in trauma‐related injuries were found in low‐resource settings by simply implementing life‐support measures by trained paramedics or nontrained community members [30, 73].

In any health system, the most important component remains the human resources, and therefore, the upscaling of those teams is an essential first step in the development of prehospital systems. In a recent study in Senegal, it was identified that the vast majority of the rural regions did not have a mature prehospital system. Interestingly, the fire department, without any health care knowledge, was responsible for prehospital patient transfers [74]. The recommendation from this study was to upscale the knowledge of the firefighters with basic lifesaving skills such as extremity hemorrhage control. This recommendation has already been implemented with qualitative feedback of successful clinical interventions in the prehospital setting (unpublished). This is just one example of the cost‐effective impact of education on health care in this rural context.

#### 4.2.4 Telemedicine

Telemedicine is increasingly being used to strengthen many components of the health care system and has proven to be effective in the prehospital system. Rural general practitioners who are not trained to provide emergency services to trauma patients may be remotely assisted by specialized practitioners [32, 42]. Examples of telemedicine use include direct supervision of procedures during resuscitation prior to interhospital transfer [72] and performing a teleultrasound with the guidance of trauma surgeons [75]. With the extensive availability of video conferencing using personal mobile devices, this strategy is an easy tool to collaboratively support patient care and safe transfer between referral and referring institutions.

#### 4.2.5 Improving Workforce, Training, and Education

The challenges of augmenting the health care workforce in rural settings include cost, difficulty in incentivizing rural living, and maintenance of competency with potentially lower volumes [8, 9, 10, 11]. As discussed previously, the education and training of existing staff in rural facilities to identify early patients requiring transfer, augment knowledge and skills in resuscitation measures, and ensure safe transfer are crucial for improved outcomes of surgical patients [40]. Additionally, Long and Sweeny (2023) suggest that providing more rural surgical training opportunities, such as a rural surgery fellowship, mentoring, and exposure in medical school, may improve the rural surgical workforce [38, 39]. To address the issue of recruitment, a market‐based approach has been suggested, which partly relies on incentivization, work/life attractiveness in rural practice, and providing opportunities to attract trainees to rural practice [38].

### 4.3 Limitations

This study is limited by dependence on available literature, which may not provide a complete understanding of the realities of health care delivery in rural contexts. However, we recognize that given the above data, there are specific strategies that are of high utility in ensuring acceptable outcomes in rural settings. In addition, only six of the OECD countries appeared in the results of this search. However, given that most of those countries are challenged by a significant rural community, we find that the conclusions drawn are relevant to the hypothesis of the paper.

### 4.4 Conclusion and Recommendations

The LCoGS made important contributions to understanding the challenges to delivering surgical care in LMICs including setting benchmarks to improve the outcomes thereof. Given that HICs face similar challenges yet maintain acceptable outcomes, we highlight that additional interventions play an important role in achieving this goal and recommend that such strategies be studied and incorporated in LMICs. These include the following:

- Regionalization and hospital designation planning.
- Education and training specifically related to early identification of patients requiring transfer, targeted resuscitation, and ensuring safe transfer. This should be extended to non‐health care staff involved in the prehospital system.
- Improved interfacility communication and support of surgical patients.

These are foundational elements of supporting rural communities and an excellent, cost‐effective, starting point to improve surgical outcomes in LMICs. A graduated approach can then be used to ultimately improve material resources to support patient transfers to higher‐level care facilities. A data‐driven process should be incorporated in parallel to inform quality assurance, improvement measures, and robust critical evaluation of such interventions.

## Author Contributions

**G. Osman:** conceptualization, investigation, writing – original draft, writing – review and editing, methodology, formal analysis, data curation. **Y. Kamel:** writing – original draft, methodology, writing – review and editing. **I. Konate:** writing – original draft, writing – review and editing. **M. Diedhiou:** writing – original draft, writing – review and editing. **S. Basnet:** writing – original draft, writing – review and editing, validation. **R. Shrestha:** writing – review and editing, writing – original draft, validation. **S. K. Shrestha:** writing – review and editing, writing – original draft, validation. **V. Krylyuk:** writing – review and editing, writing – original draft, validation. **J. Grushka:** writing – original draft, conceptualization, writing – review and editing, supervision, validation. **E. Wong:** writing – original draft, conceptualization, writing – review and editing, validation. **T. Farhat:** conceptualization, writing – original draft, writing – review and editing, methodology, validation. **K. Khwaja:** writing – original draft, writing – review and editing, validation. **D. Deckelbaum:** conceptualization, investigation, funding acquisition, writing – original draft, writing – review and editing, validation, methodology, visualization, supervision, formal analysis, resources.

## Conflicts of Interest

The authors declare no conflicts of interest.

## Data Availability

The data that support the findings of this study are available in the supplementary material of this article.
